# The effect of HIV on morbidity and mortality in children with severe malarial anaemia

**DOI:** 10.1186/1475-2875-6-143

**Published:** 2007-10-31

**Authors:** Samuel Malamba, Wolfgang Hladik, Arthur Reingold, Flora Banage, Willi McFarland, George Rutherford, Derrick Mimbe, Esau Nzaro, Robert Downing, Jonathan Mermin

**Affiliations:** 1 CDC-Uganda, Global AIDS Program, National Center for HIV, STD and TB Prevention, Centers for Disease Control and Prevention, Atlanta, GA, USA; 2 Division of Epidemiology, School of Public Health, University of California, Berkeley, USA; 3 Institute for Global Health, University of California, San Francisco, USA; 4 Pathology Department and Blood Transfusion Services, Mulago Hospital, Kampala, Uganda

## Abstract

**Background:**

Malaria and HIV are common causes of mortality in sub-Saharan Africa. The effect of HIV infection on morbidity and mortality in children with severe malarial anaemia was assessed.

**Methods:**

Children <5 years old were followed as part of a prospective cohort study to assess the transfusion-associated transmission of blood-borne pathogens at Mulago Hospital, Kampala, Uganda. All children were hospitalized with a diagnosis of severe malarial anaemia requiring blood transfusion. Survival to different time points post-transfusion was compared between HIV-infected and uninfected children. Generalized estimating equations were used to analyse repeated measurement outcomes of morbidity, adjusting for confounders.

**Findings:**

Of 847 children, 78 (9.2%) were HIV-infected. Median follow-up time was 162 days (inter-quartile range: 111, 169). HIV-infected children were more likely to die within 7 days (Hazard ratio [HR] = 2.86, 95% Confidence interval [CI] 1.30–6.29, P = 0.009) and within 28 days (HR = 3.70, 95% CI 1.91–7.17, P < 0.001) of an episode of severe malarial anaemia, and were more likely to die in the 6 months post-transfusion (HR = 5.70, 95% CI 3.54–9.16, P < 0.001) compared to HIV-uninfected children. HIV-infected children had more frequent re-admissions due to malaria within 28 days (Incidence rate ratio (IRR) = 3.74, 95% CI 1.41–9.90, P = 0.008) and within 6 months (IRR = 2.66, 95% CI 1.17 – 6.07, P = 0.02) post-transfusion than HIV-uninfected children.

**Conclusion:**

HIV-infected children with severe malarial anaemia suffered higher all-cause mortality and malaria-related mortality than HIV-uninfected children. Children with HIV and malaria should receive aggressive treatment and further evaluation of their HIV disease, particularly with regard to cotrimoxazole prophylaxis and antiretroviral therapy.

## Background

Malaria and human immunodeficiency virus (HIV) are among the leading causes of death among children in sub-Saharan Africa. In Uganda, malaria mortality among children under 5 years is 37/1,000 person-years in areas of high malaria endemicity and 18/1,000 person-years in areas of low malaria endemicity [1,2] The prevalence of HIV infection in Uganda is estimated at 7% in adults aged 15–49 years [3] and 1.7% in children under 5 years [4]. In malaria-endemic areas, repeated childhood exposure to episodes of malaria generates partial immunity, which modifies the infection but does not fully prevent parasitaemia or clinical disease [5].

Because HIV infection interferes with cellular immune function, HIV may interfere with the development of partial immunity to malaria in children. Protection against clinical malaria depends on cellular immunity, which may be impaired in HIV-infected children with low CD4 lymphocyte counts[6]. *Plasmodium falciparum *malaria lowers the CD4-CD8 ratio[7] and triggers the activation of B cells, resulting in repeated activation of T4 lymphocytes [8], a factor thought to contribute to the progression of HIV disease [9]. Furthermore, chloroquine, the most commonly used treatment for malaria in East Africa at the time of conducting the study, is itself an immunosuppressant and interferes with antigen recognition by T cells, B cell activation, and antibody production. HIV infection may also impact a child's ability to respond to malaria treatment [10,11].

Earlier studies failed to show an association between *P. falciparum *malaria and HIV infection in children [10,12-15]. However, these studies were limited by their cross-sectional nature and shortcomings in diagnosing malaria. Recent studies, including two prospective cohort studies, have shown an association between HIV-associated immuno-suppression and the severity of clinical malaria in adults. [16-20]. One prospective cohort study showed that malaria was more common among HIV-infected children <5 years old than among HIV-uninfected children[18], and a retrospective cohort study found higher mortality among HIV-infected children with malaria treated with chloroquine compared to HIV-uninfected children[21]. A prospective cohort study of patients receiving blood transfusions was conducted to examine morbidity and mortality in HIV-infected and HIV-uninfected children with severe malarial anaemia requiring transfusion.

## Methods

### Study design and subjects

This sub-study was part of a larger prospective cohort study designed to assess the risk of transfusion-associated infections among children and adults. For this analysis, study participants included children (<5 years of age) hospitalized with a diagnosis of malaria and requiring blood transfusion between December 2000 and October 2001 at Mulago Hospital in Kampala, Uganda. Mulago Hospital serves as the primary referral hospital for Uganda. Study staff identified potentially eligible subjects by reviewing all physician requests for transfusion and approached guardians or parents to determine eligibility and obtain informed consent and assent. Admission due to malaria was based on clinical criteria using sign/symptoms and the availability of a hospital bed and blood units for transfusion. Enrollment was offered immediately post-transfusion at the ward; patients were eligible for enrollment if their pre-transfusion blood specimen was available and they were residing in the greater Kampala area. Patients transfused in the six months preceding this current admission were not enrolled. Analysis was restricted to children under five years of age transfused for severe malarial anaemia whose families consented to testing the child for HIV. Investigators were not aware of the patients' HIV status, but parents/legal guardians who consented to HIV testing were counseled on their child's HIV status.

Most children were started on intravenous quinine at enrollment and prescribed sulfadoxine-pyrimethamine during follow-up. Quinine, artesunate and amodiaquine were also prescribed during follow-up, based on the child's clinical condition and anti-malarial treatment history. Patients received outpatient medical care at their follow-up visits and were referred for re-admission if warranted. The study protocol was reviewed and approved by the Institutional Review Boards of the Centers for Disease Control and Prevention and the Uganda Virus Research Institute, and approved by the Uganda National Council for Science and Technology and the University of California, Berkeley.

### Measurements

HIV and haemoglobin-testing were conducted on the pre-transfusion blood specimen obtained for blood typing and cross-matching. Information on physical examination and medical history was abstracted from patient medical records where available. Information was collected on demographic characteristics, repeat-transfusions during follow-up and common symptoms by interviewing the parent or guardian at the time of enrollment. Follow-up was for between three and six months, with clinic visits scheduled at weeks 1, 2, 4 and monthly thereafter. Follow-up visits were at the patient's bed-side in the hospital until discharge, and at the study clinic located in the hospital thereafter. A physical examination was performed at enrollment and at every other visit. An interval medical history and a blood specimen were taken at every visit. Blood smears for malaria were taken at scheduled and unscheduled follow-up visits only if the child was febrile; thus, all follow-up cases of malaria were, by definition, symptomatic malaria episodes. All care was provided free of charge, and parents were encouraged to make unscheduled clinic visits whenever their children were not feeling well. So, in most cases, parents sought medical help shortly after the time of symptom onset.

Severe anaemia was defined based on WHO adjusted criteria [5] as a haemoglobin level less than 6.1 g/dl for children less than 1 month old; less than 5.1 g/dl for children ages 1 to less than 2 months; less than 4.4 g/dl for children 2 months to less than 3 months; less than 4.7 g/dl for children 3 to less than 6 months; and less than 5.0 g/dl for children 6 months to 5-years of age. To assess mortality, field workers visited study participants' homes if they failed to appear for a follow-up visit. In addition to vital status, staff recorded reasons for loss to follow-up.

### Laboratory methods

Pre-transfusion blood specimens were screened for HIV by a single Enzyme Immunoassay (EIA) (Organon Teknica, Durham, North Carolina, USA). Serum specimens that were non-reactive on the first EIA were classified as HIV-negative; reactive specimens were retested in duplicate with a different EIA (Sanofi-Genetic Systems, Redmond, Washington, USA). If both repeat tests were non-reactive, specimens were classified as HIV-negative. If repeatedly reactive on the second EIA, specimens were confirmed as HIV-positive using Western blot (Bio-RAD). HIV infection status of children less than 24 months of age was confirmed by RT-PCR. Malaria was diagnosed using thick and thin smears and measured parasite density semi-quantitatively as the number of parasitized cells per 200 white blood cells (WBC). All malaria slides were read by two microscopists and, if the readings disagreed or for quality control purposes, the slides were read by the supervisor. The numbers of malaria parasites pre-transfusion were scored as + for 1–20, ++ for 21–250, +++ for 251–500 and ++++ for 501 and greater. Malaria was defined as reported fever and a thick smear indicating the presence of plasmodia. Hyper-parasitaemia was defined as the number of parasites per 200 WBC being greater than 20% above the study population median[22]. An episode of malaria was judged to be new if symptoms began more than 4 weeks after a previous episode. Haemoglobin was measured using the Act 5 Diff instrument (Beckman Coulter).

### Statistical methods

Based on an estimated malaria incidence of approximately 2.5% per 6 months in the unexposed group, and a type I error of 0.05, it was estimated that a cohort of 850 individuals would have 80% power to detect an incidence rate ratio of 1.16 or a 16% increase in the malaria incidence rate in the exposed group. Data were double-entered using Epi-Info (CDC 2075A West Park Place, Stone Mountain, GA 30087 USA) and analysed using STATA (Stata Corporation, 702 University Drive East, College Station, Texas 77840 USA).

Baseline differences were compared between HIV-infected and HIV-uninfected children with respect to age, sex and severity of malaria on admission. The likelihood of death occurring within 7, 14, 21 and 28 days following a diagnosis of an episode of symptomatic malaria was evaluated. Cox proportional hazards models were used to compare mortality rates between HIV-infected and HIV-uninfected children, with and without adjustment for baseline differences in malaria treatment, age and sex. The proportionality assumption was tested using log-log plots, and generalized estimating equations (GEE) were used to analyze repeated measurement outcomes of hospital re-admission, new malaria episodes during follow-up.

## Results

Data were analysed from 847 children with a median age of 1.2 years (inter-quartile range [IQR]: 0.7, 2.0). At enrollment, HIV-infected children (n = 78, 9.2%) did not differ from HIV-uninfected children (n = 769) with respect to sex, age, pre-transfusion hyper-parasitaemia and others markers of severity of malaria (Table 1). A total of 803 children (95%) completed follow-up; 18 (2%) were lost to follow-up, 13 (1.5%) moved before completing follow-up, and 13 (1.5%) refused further follow-up. The median follow-up time was 162 days (IQR: 111–169). The total follow-up time was 23.6 person-years for the HIV-infected and 281.3 person-years for the HIV-uninfected children.

**Table 1:** Sex, age and markers of severity of malaria at enrollment among HIV-infected and HIV-uninfected children under 5 years, Kampala, Uganda

Baseline variables	HIV- uninfected children (n = 769)		HIV-infected children (n = 78)		Total N = 847		P-value*
Sex (female)	356	46%	37	47%	393	46%	0.85
Age under 1 year	321	42%	30	38%	351	41%	0.58
Respiratory distress	287	37%	30	38%	317	37%	0.84
Jaundice	71	9%	6	8%	77	9%	0.64
Splenomegaly	388	52%	41	53%	429	52%	0.87
Hepatomegaly	160	21%	15	19%	175	21%	0.68
Post-transfusion severe anaemia^+^	494	78%	51	80%	545	78%	0.78
Pre-transfusion hyper-parasitaemia	55	7%	6	8%	61	7%	0.82
Treatment with Quinine (I.V.)	584	76%	57	74%	641	76%	0.57

*Chi-square test for difference in proportions

+Enrollment haemoglobin levels available for 632 HIV-uninfected children and 66 HIV-infected children

### Mortality

Vital status was determined at the last date of follow-up for all 847 children. Seventy-six children (9.0%) died, including 26 (33.3%) HIV-infected children and 50 (6.5%) HIV-uninfected children. Of the 76 deaths recorded during follow-up, 45 (59%) occurred within 28 days of enrollment. During the 28 days following enrollment, HIV-infected children were more likely to die than HIV-uninfected children (hazard ratio (HR) 3.70 (95%CI 1.91–7.17; *P *< 0.001). The corresponding age, sex and anti-malarial treatment adjusted hazard ratios of dying within 7, 14, 21 days of a malaria episode between HIV-infected children and HIV-uninfected children were 2.86 (95% CI 1.30–6.29, P = 0.009), 3.13 (95% CI 1.54–6.37, P = 0.002) and 3.46 (95% CI 1.74–6.87, P < 0.001), respectively.

During the six months following enrollment, the survival probability was significantly worse among HIV-infected children compared to HIV-uninfected children (log-rank test for equality of survivor functions: χ^2 ^= 65.9, p < 0.001). In the Cox proportional hazards analysis, HIV-infected children with severe malarial anaemia had a significantly higher rate of all-cause mortality (HR = 5.70, 95% CI 3.54 – 9.16, P < 0.001) and of death occurring within 7, 14, 21 or 28 days of a symptomatic malaria diagnosis (HR 3.74 (95% CI 1.93 – 7.26, P < 0.001), 3.87 (95% CI 2.09 – 7.17, P < 0.001), 4.16 (95% CI 2.28 – 7.60, P < 0.001) or 4.88 (95% CI 2.83 – 8.42, P < 0.001), respectively) when compared to HIV-uninfected children (Table 2).

**Table 2:** Morbidity and mortality among HIV-infected and HIV-uninfected children <5 years old with severe malarial anaemia

	**HIV infected children (N = 78) **Years of follow-up = 5.2		**HIV uninfected children (N = 769) **Years of follow-up = 55.0		† Hazard ratio (HR) or Incidence rate ratio (IRR)	**P-value**
**In the 28 days following first admission with severe malarial anaemia**	Episodes or Deaths	Rate per 100 person-years	Episodes or Deaths	Rate per 100 person-Years		

**Mortality**						
Death within 7 days of a malaria episode	8	153.8	28	50.9	2.86 (1.30 – 6.29)	0.009
Death within 28 days of a malaria episode	12	230.8	33	60.0	3.70 (1.91 – 7.17)	<0.001
**Hospital admissions**						
Any reason for re-admission	11	211.5	28	50.9	4.05 (1.95 – 8.43)	<0.001
Re-admission due to malaria	5	96.2	13	23.6	3.74 (1.41 – 9.90)	0.008

**In the 6 months follow-up **(Includes repeat episodes)						

**Mortality**						
All cause mortality	26	110.2	50	17.8	5.70 (3.54 – 9.16)	<0.001
Death within 7 days of any malaria episode	12	50.8	33	11.7	3.74 (1.93 – 7.26)	<0.001
Death within 28 days of any malaria episode	19	80.5	41	14.6	4.88 (2.83 – 8.42)	<0.001
**Hospital admissions**						
Any reason for re-admission	18	76.3	66	23.5	2.93 (1.62 – 5.29)	<0.001
Re-admissions due to malaria	7	29.7	26	9.2	2.66 (1.17 – 6.07)	0.02
**Malaria incidence**						
Malaria diagnosed during visits after enrollment	92	394.1	881	313.9	1.12 (0.91 – 1.38)	0.273
Malaria episode >28 days after last malaria episode	54	228.8	582	206.9	0.98 (0.76 – 1.28)	0.895
**Unscheduled visits**						
Unscheduled visits to the clinic	24	101.7	227	80.7	1.14 (0.74 – 1.77)	0.543

† Adjusted for age, sex and baseline quinine treatment status

Though not statistically significant, high density malaria episodes (hyper parasitaemia) were associated with an increased risk of dying within 7 days of a symptomatic malaria diagnosis. This risk of dying in children with high density malaria episodes was approximately two times that of children with low parasite density, HR = 1.62 (95% CI 0.22 – 12.02, P = 0.64) for HIV-infected and HR = 2.25 (95% CI 0.90 – 5.61, P = 0.08) for HIV-uninfected children.

### Malaria infections and unscheduled clinic visits

*P. falciparum *was the only infecting malaria species in all the samples tested. No difference was found in the incidence of diagnosed malaria during visits in the six months of follow-up between the HIV-infected and uninfected children, even after restricting analysis to malaria episodes that occurred more than 28 days apart (IRR 0.98, 95% CI 0.76 – 1.28). There was no difference in the incidence of unscheduled clinic visits between HIV-infected and uninfected children (Table 2).

### Morbidity: Re-admission following an initial episode of severe malarial anaemia

Thirty-six children (4%) were re-admitted to hospital within four weeks following transfusion for malarial anaemia, including 10 (13%) of the HIV-infected and 26 (3%) of the HIV-uninfected children. The risk of hospital re-admission due to malaria within 28 days following a first admission with severe malarial anaemia, adjusted for differences in age and anti-malarial treatment received before enrollment, was substantially elevated (IRR 3.74, 95% CI 1.41 – 9.90) in the HIV-infected children compared to HIV-uninfected children. During the 6 months of follow-up, re-admission due to malaria was also more common (IRR 2.66, 95% CI 1.17 – 6.07) in HIV-infected children than in HIV-uninfected children (Table 2).

## Discussion

In children less than five years of age transfused for severe malarial anaemia, restricting the analysis to 28 days after enrollment, HIV-infected children were approximately three times more likely to die within seven days of a symptomatic malaria episode than HIV-uninfected children. The increased risk of mortality remained, even after adjusting for age, sex, malaria treatment regimen and clinical signs of severe malarial anaemia at enrollment. The mortality findings are consistent with longitudinal studies in adults [23,24] and a retrospective study in children[21]. In another study conducted in Uganda [16], HIV-infected children had more hospitalizations but showed no difference in the incidence of malaria episodes when compared to HIV-uninfected children.

A causal relationship between HIV-infection and poor response to malaria treatment is theoretically consistent with what is known about HIV pathogenesis [7,8]. HIV-infected children had similar rates of malaria episodes but were hospitalized more often than HIV-uninfected children. This finding suggests that HIV-infected children may have an impaired immune response, impeding clearance of malaria parasites and increasing the risk of complications that may require hospitalization.

A limitation of this study was the uncertainty as to whether the excess deaths among the HIV-infected children with severe malarial anaemia were due to malaria or due to other competing causes related to HIV infection. Without a post-mortem examination, it is difficult to ascertain the cause of death and eliminate competing causes of death due to other HIV associated co-morbidities. However, an association was found between HIV infection and mortality even when the outcome measure was restricted to deaths occurring within seven days of diagnosis of severe malarial anaemia, making competing illnesses a less likely explanation. Data on clinical staging or CD4 count for HIV-infected children were not available. CD4 cell count has previously been associated with an increased risk of malaria infection [25], and a causal relationship between HIV-related immunosuppression and malaria-related mortality would be strengthened by observing an effect between lower CD4 count and increased mortality. Differences in anti-malaria treatment received before admission and during follow-up might have affected the risk of mortality; however, because 75% of study participants were treated with quinine at baseline and both HIV-infected and HIV-uninfected children were treated using the same anti-malarial treatment protocol during follow-up, it is unlikely that treatment differences in this study significantly affected the final results. None of the study participants was on antiretroviral therapy or cotrimoxazole prophylaxis at the time of the study. Without data from blood and other cultures on treatments such as antimicrobial therapy it was not possible to rule out the possibility of co-existing bacteraemia, which is a common cause of morbidity and mortality in children in Uganda. Bias could also have been introduced if parents/legal guardians counseled on their child's HIV status were more likely to bring their children for health care during follow-up and therefore leading to an increase in hospital admissions. However, when hospital admission rates were compared between HIV-infected children whose parents/legal guardians knew their child's HIV status and those who did not know their child's HIV status, no significant difference was observed [Data not shown]. Lastly, the occurrence of haemoglobinopathies, in particular Glucose 6-phosphate (G6P) dehydrogenase deficiency may pre-dispose HIV-infected children to more recurrent anaemia and therefore modify the effect of HIV on the incidence of malaria.

The interaction between malaria and HIV-infection among children has profound implications for child health in Africa, given the high incidence of malaria and the fact that more than 90% of the world's children with HIV reside in Africa. Several interventions regarding malaria may be beneficial for HIV-infected children living in Africa. Cotrimoxazole prophylaxis has been associated with a reduced incidence [26,27] of malaria and lower mortality [26] among HIV-infected children, and is currently recommended by WHO[28]. Insecticide-treated bed nets are associated with a 50% reduction in malaria among children living in endemic areas[29], and are likely to be equally effective for HIV-infected and uninfected children. This study has demonstrated that children infected with HIV and malaria have more severe malaria disease outcomes and therefore should receive aggressive treatment and further evaluation of their HIV disease outcomes for a possibility of starting them on antiretroviral therapy. Additionally, other basic interventions to prevent malaria should be initiated in HIV-infected children. In particular, consideration should be given to initiating cotrimoxazole prophylaxis while the HIV/AIDS disease status is being determined and providing parental education regarding febrile episodes in their HIV infected children.

## Authors' contributions

Concept

Samuel Malamba, Wolfgang Hladik, Esau Nzaro, Jonathan Mermin

Design

Samuel Malamba, Wolfgang Hladik, Flora Banage, Esau Nzaro, Robert Downing, Derrick Mimbe, Jonathan Mermin

Analysis

Samuel Malamba, Arthur Reingold, Jonathan Mermin

Writing/Revising manuscript

Samuel Malamba, Arthur Reingold, Wolfgang Hladik, Willi McFarland, George Rutherford, Robert Downing and Jonathan Mermin led the writing of the manuscript, though all authors participated in reviewing and revising the manuscript
